# Robust Financial Fraud Alerting System Based in the Cloud Environment

**DOI:** 10.3390/s22239461

**Published:** 2022-12-03

**Authors:** Branka Stojanović, Josip Božić

**Affiliations:** Joanneum Research, DIGITAL—Institute for Information and Communication Technologies, 8010 Graz, Austria

**Keywords:** fintech, fraud detection, cloud security, risk assessment, formal model checking, machine learning, anomaly detection

## Abstract

The digitalisation of finance influenced the emergence of new technological concepts for existing user needs. Financial technology, or fintech, provides improved services for customers and new economic value for businesses. As such, fintech services require on-demand availability on a 24/7 basis. For this reason, they are often deployed in cloud environments that allow connectivity with ubiquitous devices. This allows customers to perform online transactions, which are overseen by the respective financial institutions. However, such cloud-based systems introduce new challenges for information security. On one hand, they represent attractive targets for cyberattacks. On the other, financial frauds can still go unnoticed by the financial institutions in charge. This paper contributes to both challenges by introducing the concept for a cloud-based system architecture for fraud detection and client profiling in the banking domain. Therefore, a systematic risk assessment was conducted in this context, and exploitation probabilities were inferred for multiple attack scenarios. In addition, formal verification was accomplished in order to determine the effects of successful vulnerability exploits. The consequences of such security violations are discussed, and considerations are given for improving the resilience of fintech systems.

## 1. Introduction

The term fintech was introduced in 1972 and denotes the intersection of finance with information technology [1]. As such, it represents an umbrella term for virtually connected technologies for financial services. Since then, fintech has gained much popularity among its users, and this trend is expected to continue in the future [2,3]. Services such as digital banking and payments are made possible by the advancements in the finance sector. However, in order to deploy fintech applications, their providers rely on an appropriate technological infrastructure. For this reason, fintech solutions can make use of benefits from the domain of cloud computing. Cloud solutions offer processing capabilities for massive amounts of data, but also increased security and availability [4]. For example, cloud service providers such as Microsoft Azure [5] offer hosting solutions to financial institutions and their clients. Additionally, the advance of ubiquitous computing allows users to access cloud services from a wider range of devices. In the context of fintech, this implies that organisations, cloud providers and their clients can exchange and process large amounts of financial data in real time.

Unfortunately, financial fraud also represents an issue for cloud-based fintech systems. For example, criminal acts such as credit card information theft happen on a daily basis and in large numbers [6]. This fraud compromises legitimate credit card owners and causes financial and other forms of damage. Therefore, detecting fraudulent behaviour and tracking down malicious users represents a serious security challenge. Usually, anomaly detection techniques from machine learning (ML) are applied to identify suspicious behaviour patterns in payment datasets. In this way, ML provides predictions from available payment data and helps in further decision making [7]. Another problem for cloud and fintech systems is cyberattacks, which can violate information security or disrupt the functionality of a system [8]. Additionally, AI has become a target for so-called adversarial attacks in recent years [9]. For these reasons, ensuring security and privacy in the ubiquitous environment of fintech is of uttermost importance [10,11].

The contributions of this paper are twofold: It introduces a cloud-based system architecture that serves as a representation of a fraud alerting system. This constituent part of a theoretical banking system is used to tackle the above security challenges. The conceptual system comes in two versions and relies on existing ML techniques. First, a systematic risk assessment is conducted to identify cyber threats that might occur in the system. Then, several cyberattack scenarios are defined, which are supported by their exploitation probability based on several metrics. The second challenge is the identification of malevolent users who had committed acts of financial fraud. In particular, a fintech component is introduced, whose task is to automatically detect credit card fraud in financial transactions. The proposed solution uses a ML-based scoring system that analyses user behaviour in fintech transactions. Finally, formal methods are applied to provide exposure assessment with the help of a state-of-the-art model checker. It is important to note that cyberattacks against the proposed system do not correlate with the emergence of financial frauds in the first place. However, an attacker can influence the functionality of the scoring system and cause false indications of fraud.

The remainder of the paper is structured as follows: First, Section 2 enumerates existing works related to the mentioned challenges. Then, Section 3 proposes the concept for a fraud alerting system. Section 4 describes the formal modelling methodology, and Section 5 elaborates on the respective risk assessments of the systems. Afterwards, Section 6 discusses the results, and Section 7 concludes the paper.

## 2. Related Work

Several works exist that analyse cybersecurity in the emerging domain of cloud computing. In many cases, the focus lies on the application of existing tools and techniques to cover this issue. These papers describe frameworks to execute cyberattacks in simulated cloud environments and discuss resulting observations. In addition, the authors conducted risks and resilience assessments in this environment and discussed the impacts of attacks in the context of their technological solutions. A comparative table is given in Table 1, where each reference is categorised according to four main topics. These are the assessment of risks and resilience, applied AI techniques, formal modelling and verification and cybersecurity. In addition, the contribution of each paper is given.

The authors of [12] conducted a risk assessment for attacks in a cloud infrastructure. Therefore, they identified risks for information security in the system’s assets and their relations. Subsequently, the identified vulnerabilities are prioritised with the Common Vulnerability Scoring System (CVSS) [13], thereby providing indications on the implementation of protection mechanisms.

The work in [14] addresses the existing gap between impact assessments of cyberattacks and cyber resilience in the cloud environment. The difficulty of conducting cyber resilience in this environment represents an issue due to the sheer amount of different aspects. For this reason, they present a framework to depict impacts of cloud-level attacks. A metric is introduced in order to assess security of network configurations, which is calculated in CVSS by consulting expert knowledge.

In [15], the authors propose a platform which is designed to reproduce realistic cybersecurity scenarios in a virtual environment. The simulation reflects real cloud systems that reproduce realistic benign and malicious human behaviour. In this way, the platform enables the assessment, teaching and learning of potential security-related issues. For this sake, two use cases are defined that contain several typical types of cyberattacks. Subsequently, the outcome of the simulation is produced in form of system logs that can be analysed to track malicious behaviour and software.

The authors of [16] propose a framework to tackle security issues in cloud computing devices with regard to user privacy. Their framework applies different technologies to prevent cyberattacks by identifying suspect edge devices. The framework was tested with typical attacks in a virtual environment that relies on Microsoft Azure. Eventually, it produces a log repository that contains information on identified malicious activity.

In [17], a cyberattack simulation was used to assess security in network systems. The application represents probabilistic modelling and simulation language for modelling a Microsoft Azure cloud infrastructure. Attacks are simulated for system-specific scenarios that are executed in a step-wise manner. As such, the simulation can be tracked from the system entry point to various attacks paths in the model. Subsequently, each step can be analysed to point to weaknesses for each asset.

The work in [18] analyses cyberattacks in a cloud-based smart farm infrastructure. The smart farm relies on the Microsoft Azure services and communicates over the IEEE 802.11 protocol. The authors emulated a concrete DoS attack in this context, namely, the Wi-Fi deauthentication attack.

The paper [19] discusses best practices for protecting the users of a public cloud-based environment. Thus, strategies, tools and guidelines are discussed, but the work also provides a description of how to apply defence postures in conjunction with best practices from the industry. By doing so, it consults the MITRE ATT&CK knowledge base [20] and addresses the challenges for cyberattacks in cloud systems.

The authors of [21] introduced a framework for modelling security dependencies in the form of attack graphs. The probability of vulnerability exploitation is calculated according to the base score from CVSS for each note at an attack path.

The paper [22] addresses risk assessment in ubiquitous environments with a focus on cloud computing. For this reason, the authors formulated a methodology that contains a multi-attacker and multi-target graphical model to conduct the assessment. In their proposed scheme, the model considers information such as system configuration, vulnerability details and CVSS information. They used a theoretical example of a smart airport network where information is exchanged dynamically with a cloud system. As part of the vulnerability-based assessment, a greedy strategy is proposed for the reduction of risk likelihood at edge devices. In this way, attack paths are identified in the graphical model in order to reduce the risk values.

The work in [23] addresses existing cybersecurity issues in cloud environments by applying formal verification. The proposed solution defines security properties by addressing cyberattacks in online environments. In this approach, a formal model of the system’s architecture is made by focusing at its critical components. Subsequently, formal verification is applied to check the system’s consistency with predefined requirements.

In [24], a formal model and security properties of a system are provided. In addition, the authors applied model checking of the defined properties with respect to the expected functionality. The obtained results also enabled them to test the system in a model-based manner. Similarly to our approach, several main components and several security properties were defined in a formal model. Then, model checking was applied to verify that a malicious actor can exploit data integrity and confidentiality.

In addition, an extensive survey on AI solutions for security threats is given in [25]. There, a taxonomy of cyber vulnerabilities is given in the context of, among others, cloud-based scenarios. Then, AI methods are discussed with respect to their applicability to addressing existing security challenges. Therefore, different types of threats are discussed, including cyberattacks such as DoS attacks and other violations of information security.

An overview of cyber threats in the cloud environment can be found in [26]. In addition, the paper in [27] provides a survey on cyberattacks in IoT-based cloud computing. Systematic surveys on adversarial attacks against ML are given in [9,28]. Security and privacy considerations for the domain of ubiquitous computing are given in [29]. In general, the application of formal models and model checking in cyber security is reviewed in [30]. Additionally, broader cybersecurity issues on the organisational and management level with regard to standards in cloud computing are given in [31].

**Table 1:** Comparative table with potential applications.

Ref	Risk/Resilience Assessment	AI/ML	Formal Models Verification	Cyberattacks	Potential Contribution
[12]	X				Application to fintech and fraud detection domain.
[14]	X			X	Additional risk factors can be incorporated into the framework.
[18]				X	Application to fintech and fraud detection domain and consideration of additional cyberattacks.
[19]	X			X	User scoring systems can be implemented in a cloud environment and use the official support.
[21]	X	X		X	Cyberattacks against the cloud can be modelled in attack graphs.
[22]	X	X		X	User scoring systems can be used for vulnerability-based assessment.
[32]	X		X	X	Real-world cloud system concept that can be used in analysis and implemented.
[23]			X	X	Additional cloud-related security issues in form of a greater challenge.
[24]			X		System models to generate and execute model-based tests.
[25]		X		X	AI solutions can be applied to scoring systems.

## 3. Proposed Solution

This section describes the methodology used and the proposed solution for a robust financial fraud alerting system.

The main objective of this work includes a proposal of two system concepts and potential concepts’ implementations in a cloud service. It also includes an extensive analysis and comparison of two proposed concepts, which is based on formal modelling. This analysis covers two aspects—(i) the analysis of the fraud-detection reliability of the implemented concept, and (ii) the analysis of the concepts of implementation security and resilience in a cloud environment, including vulnerabilities and potential cyberattack analysis.

**Figure 1:**
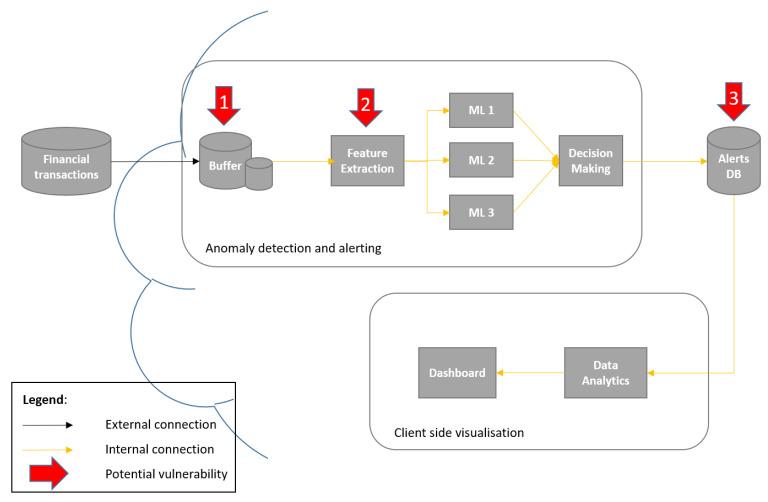
Financial fraud alerting system based on parallel anomaly detectors.

**Figure 2:**
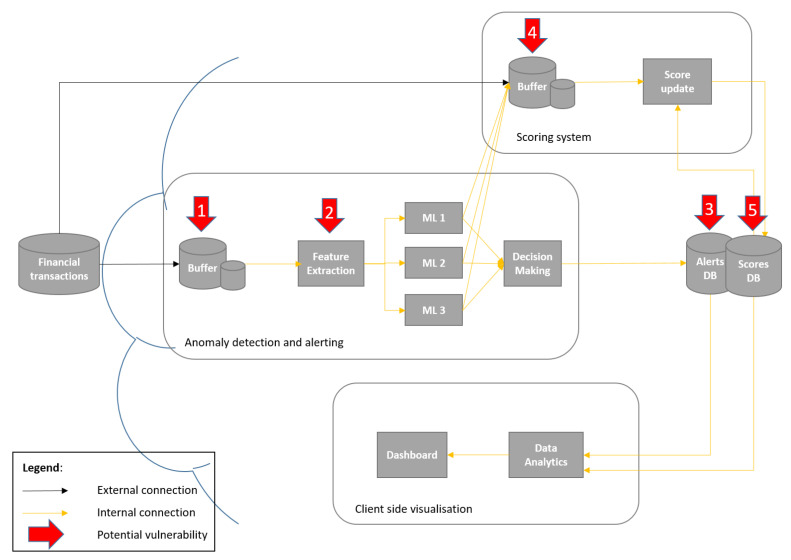
Machine-learning-based scoring module architecture.

The system itself represents a cloud service that contains two distinctive modules: One module handles fraud detection within financial (credit card) transactions, whereas the other is responsible for fraud alerting (Figure 1 and Figure 2). It should be noted that fraud detection is based on different ML anomaly detection techniques, which were previously proposed and extensively evaluated in our previous work in [33]. This means that they will not be re-evaluated in this work. On the other hand, two different concepts for implementation of an alerting module are proposed, evaluated and compared in this paper—namely, (1) the basic financial fraud alerting system based on parallel anomaly detectors, and (2) the extended alerting system, which includes a ML-based scoring module as an additional alerting component.

The novelty of this paper is reflected in several aspects:

- Fraud detection robustness—ensured through the proposed parallel processing anomaly detectors;
- Alerting robustness—a novel scoring system is proposed, representing the users’ historical behaviour analysis;
- An extensive cyber resilience analysis—the system’s robustness during different cyberattacks targeting the cloud infrastructure, analysed by using formal methods.

### 3.1. Methodology

The work in this paper reflects the partial results of a research project which deals with developing a ML-based cloud service for fintech fraud detection. The complete research pipeline in this project includes two logical and practical units which cover different aspects of the problem:

1. **Part 1**: Investigating and testing of different ML-based anomaly detection techniques for fraud detection in the fintech environment. This part was proposed and extensively reported in our previous paper in [33], which included the following aspects:
  - Extensive SoA analysis covering automated fraud detection in the credit card , financial transactions and blockchain fintech domains;
  - Analysis and case studies’ definitions based on publicly available datasets in this domain, including credit card fraud detection (CreditCard dataset), financial transactions fraud detection (PaySim dataset) and bank transactions fraud detection (BankSim dataset);
  - Investigation and testing of suitable preprocessing techniques, including statistical analysis, feature engineering and feature selection based on information value;
  - Analysis and testing of different applicable ML techniques, including outlier detection methods (local outlier factor, isolation forest and elliptic envelope) and ensemble approaches (random forest, adaptive boosting and extreme gradient boosting);
  - Reliability analysis of anomaly detection algorithms based on layer-wise relevance propagation.
2. **Part 2**: Resilience analysis of the system’s implementation possibilities in the cloud environment. This part presents the main focus of this paper. It involved findings and partial results from Part 1 and performance of a resilience analysis of implementation possibilities for the fraud detection service in the cloud environment. We also propose an additional scoring-based alerting logic which uses historical decisions of the ML models. It includes following aspects:
  - SoA analysis covering risk and resilience assessment, formal verification and cybersecurity in cloud environments;
  - Case study selection based on case studies in Part 1—credit card fraud detection (CreditCard dataset) based on outlier detection methods (local outlier factor, isolation forest and elliptic envelope);
  - The fraud detection robustness is improved through the proposed implementation that includes parallel processing anomaly detectors;
  - Alerting robustness is improved by proposing a novel ML-based scoring system representing the users’ historical behaviour analysis;
  - The system’s resilience is analysed during different cyberattacks targeting the cloud infrastructure by using formal methods.

It has to be noted that this paper, as it covers the second part of the research pipeline described above, does not include any additional analysis, training or testing of ML-based fraud detection techniques. As such, it utilises the findings from the selected case study from our previous work in [33] as inputs for the conducted formal probabilistic analysis.

### 3.2. Financial Fraud Alerting System Based on Parallel Anomaly Detectors

The proposed basic fraud alerting system, as depicted in Figure 1, presents a cloud service that consists of three main modules: (i) anomaly detection and alerting module, (ii) alerts database and (iii) client side visualisation module. (Please note that numbers on the red arrows indicate attack scenarios that are described in more detail in Section 5.2). The main contributions of this paper are focused on the anomaly detection and alerting module, and the client side visualisation module is only considered as a necessary component in such a service, and is not specifically addressed in the following description and experiments.

The anomaly detection and alerting module is based on ML, and consists of some common components, such as a buffer, a feature extractor and trained ML-based anomaly detectors. Further, the proposed system utilises three different parallel anomaly detectors, instead of a commonly used one, and proposes a rule-based decision-making component. The input for one execution of the anomaly detection and alerting service is a feature vector (i.e., data), which represents a financial transaction originating from a certain user and at a given moment. Successively, the output represents a binary alert, which indicates a suspected fraudulent transaction.

#### 3.2.1. Anomaly Detection

The focus of the conducted experiments in the context of the proposed system was not to depict the best ML-based algorithm but to formally check the performance of such a system, including:

- Formal checking of the influence of additional parallel detectors, with regard to true positive rate (TPR) and true negative rate (TNR), during the system’s functioning with uninterrupted by cyberattacks;
- Formal checking of the system behaviour and the influence of different cyberattacks on the system’s performance, reflected in missed alerts (false negatives) and false alerts (false positives).

It should be noted that the considered cyberattacks include different malicious activities that aim for the cloud infrastructure of the system. The potential financial frauds are separately considered, namely, in the form of modelled (expected) user behaviour. Figure 1 presents also potentially vulnerable system components in the case of a cyberattack, but this will be discussed in more detail in Section 5.

As already mentioned, the basis for this work is our previous research in [33], where we proposed and tested several ML methods. The main goal was to detect fraudulent financial transactions by using several publicly available datasets. However, one of the biggest challenges in that paper was the lack of publicly available datasets. This was mostly due to privacy issues, considering that datasets can contain sensitive and personal data. Three publicly available datasets were identified as suitable and not outdated:

- CreditCard: This credit card fraud detection dataset contains transactions made with credit cards of European cardholders in September 2013 (encoded as PCA components).
- PaySim: Synthetic financial datasets for fraud detection; the authors of this dataset used aggregated data from a private dataset to generate a synthetic one. The synthetic dataset resembles the common operation of transactions, but contains injected malicious behaviour to be able to evaluate the performance of fraud detection methods.
- BankSim dataset: Synthetic data from a financial payment system. In order to generate this dataset, its authors used an agent-based simulator of bank payments. This was based on a sample of aggregated transactional data that was provided by a bank in Spain.

Considering that only the CreditCard dataset contains representations of the real transactions, and thus represents the most credible data reflecting user behaviour, we decided to use the statistics of that dataset as inputs for probabilistic model checking. This dataset contains 492 fraudulent transactions out of 284,807 transactions within two days. However, this makes the dataset highly unbalanced, since the positive class’s percentage (fraud) of all transactions equals 0.172%.

We also decided to use our outlier detection methods from [33] as the methods of choice for the further consideration for parallel anomaly detectors, namely: ML1—local outlier factor, ML2—isolation forest and ML3—elliptic envelope. The performance that was achieved by these methods on the CreditCard dataset was taken as an input for formal modelling in the following manner:

- True positive rate (TPR) represents the probability that fraudulent activity (transaction) will be correctly detected;
- True negative rate (TNR) represents the probability that a regular (non-fraudulent) transaction will be correctly classified.

True positive and true negative rates for all three proposed methods are shown in Table 2.

#### 3.2.2. Decision Making

The decision-making component in the proposed system represents a simple rule based decision-making mechanism. This gives the positive output—referred to here as $Alert_{anomaly}\left(t\right)$, at the moment *t* if at least two out of three detectors identified the anomaly. The logic of the applied decision-making is presented in the following equation.

(1) $$Alert_{anomaly}\left(t\right)=\left\{\begin{array}{cc} 1, & \frac{ML1(t)+ML2(t)+ML3(t)}{3}\geq \frac{1}{2}\\ 0, & \;\mathrm{otherwise} \end{array}\right.$$

where,

$$\begin{array}{c} ML_{X}\left(t\right)=\left\{\begin{array}{cc} 1, & \;\mathrm{suspected}\;\mathrm{fraudulent}\;\mathrm{transaction},\\ 0, & \;\mathrm{regular}\;\mathrm{transaction}. \end{array}\right. \end{array}$$

### 3.3. System Extension—Machine-Learning-Based Scoring Module

The extended variant of the proposed fraud alerting system, in addition to the module described in the previous subsection, includes a ML-based scoring module as an additional alerting module. This module considers historical user data, and its main purpose is to increase the resilience of the alerting system as a whole. Figure 2 depicts the architecture of the extended fraud alerting system, including the (i) anomaly detection and alerting module, (ii) scoring system module, (iii) database with two instances/services (alerts database and scores database) and (iv) client side visualisation module. (Please note that numbers on the red arrows indicate attack scenarios that are described in more detail in Section 5.2). Similarly to the basic fraud alerting service, Figure 2 depicts also potentially vulnerable system components in the case of a cyberattack (see Section 5).

The scoring system consists of a buffer and the score update decision component, and takes as inputs ML model outputs and the history data stored in a special instance of the service database—scores DB. While the output of the anomaly detection and alerting component is calculated with the same logic as the basic fraud alerting system, $Alert_{anomaly}\left(t\right)$, (Equation (1)), the output of the scoring module is an additional alert, referred to here as $Alert_{score}\left(t\right)$. This alert takes into account, besides the current transaction, the previous two historical outputs of the ML-based anomaly detectors for the same user, stored in the system database (scores DB). The logic behind the score alert is presented in the equations below.

(2) $$Alert_{score}\left(t\right)=\left\{\begin{array}{cc} 1, & \frac{ML_{avg}\left(t\right)+ML_{avg}\left(t-1\right)+ML_{avg}\left(t-2\right)}{3}\geq \mathrm{threshold}\\ 0, & \;\mathrm{otherwise} \end{array}\right.$$

where,

$$\begin{array}{c} ML_{avg}\left(t\right)=\frac{ML1(t)+ML2(t)+ML3(t)}{3}. \end{array}$$

In this work, two threshold values were tested in the experiments in order to test the influence on rising the alerts. The first one is the arithmetic average (threshold=1⁄2), whereas the choice of second one was inspired by the fact that we used three parallel detectors. As such, it was set to threshold=2⁄3.

Both alerts, namely, $Alert_{anomaly}$ and $Alert_{score}$, are sent in parallel to the client-side visualisation module and made available to the user of this service. This was done regardless of whether this was the transaction’s originator or the responsible actor.

## 4. Assessment of Cyber Resilience Using Formal Methods

Formal modelling poses one of the methods to detect weaknesses and possible vulnerabilities at an early stage of system design. As such, it is commonly applied during the process of hardware and communication protocol design. However, formal methods also found their application in the risk and resilience analysis. In this domain, probabilistic formal modelling tools are used in the most cases.

This section describes the methodology for the risk and resilience analysis proposed in this work. In addition, it provides an overview of the applicable formal methods.

### 4.1. Resilience Analysis Work-Flow

**Figure 3:**
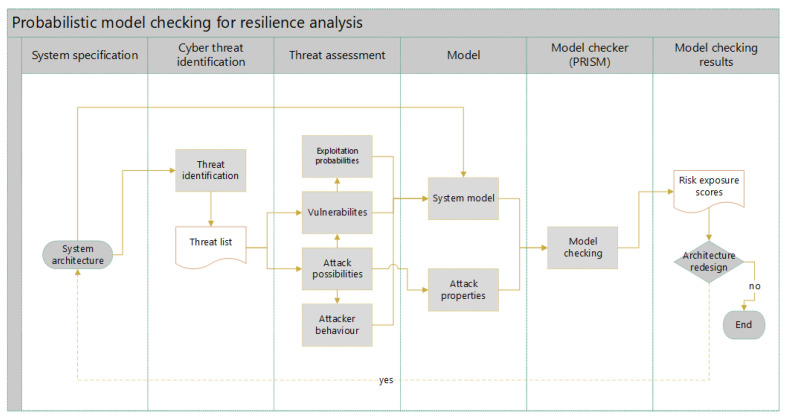
Proposed methodology for the resilience analysis based on probabilistic model checking.

The flowchart of the methodology proposed in this paper is given in Figure 3. The depiction reflects the risk and resilience analysis based on formal methods, i.e., probabilistic model checking. For this sake, we selected the PRISM model checker [34] for formal verification in this work. The reasons for this choice are explained in the next subsection. The steps in the process are logically grouped in several categories:

1. System specification: Definition of the system’s architecture, its components and communication channels.
2. Cyber threat identification: In order to identify cyber threats in the proposed systems, its technological components need to be considered separately. In fact, threats are identified with respect to the system’s architecture—those which are typical for each component. For this reason, personal expertise and available information sources (e.g., [20]) were consulted to infer a list of threats for both proposed systems.
3. Threat assessment: According to the obtained list of threats and the system’s architecture, possible entry points for an attacker are identified. Then, cyber vulnerabilities are attributed to certain entry points for an attacker and result in different types of exploits. Subsequently, corresponding attack scenarios are defined that depict the behaviour of an attacker in the given environment. For every vulnerability, exploitation probabilities are calculated according to several metrics, which each represents the likelihood of occurrence for a given attack.
4. Model: The formal model was created in the PRISM modelling language based on the proposed architecture; we identified vulnerabilities with exploitation probabilities and modelled attacks. The non-deterministic model was developed as a Markov decision process (MDP). Formal attack properties were identified and modelled next using the probabilistic computation tree logic (PCTL) embedded in the PRISM model checker.
5. Model checker (PRISM): Model checking was performed against the identified properties using the PRISM model checker. The required input elements for formal verification were the system model and the identified attack properties.
6. Model checking results: This process resulted in the maximum likelihoods of successful attack attempts—risk exposure scores.

Modelled input in our PRISM model is a state of the input transaction (1—fraudulent, 0—regular), where we define the probability of a transaction being fraudulent based on the case study (the CreditCard dataset used in our case study has the ratio of fraudulent transactions of 0.172%). All system components are modelled as modules in PRISM modelling language. Every module can change the inner state of our transaction; e.g., there is a probability of 0.8824 that ML1 is going to detect a fraudulent transaction (and leave it in the state 1 for the processing by the next module), and consequently a probability of 0.1176 that the transaction is going to be misclassified as regular (and its state is going to be changed to 0). Apart from the uncertainty that ML algorithms limitations bring to the alerting process, certain cyber-attacks (unrelated to the fraud originator) can also influence the alerting process (and cause changing of the state of our transaction in the model). The PRISM model checker checks the probability of every possible path in the defined system and calculates risk-exposure scores for the defined attack properties. For example, we model as an attack property that the client-side visualisation service is going to receive the information that the state of the transaction is 0 (regular), under certain conditions (active/no cyber-attacks), while the input transaction was in the state 1 (fraudulent).

### 4.2. Overview of Formal Methods

Different parts of a system, including the functional correctness of implementations, programming bugs, hardware Trojans and security properties, can be formally checked using formal methods. In general, they can provide both qualitative and quantitative analysis [32,35,36]. These aspects are covered by a variety of formal verification tools. While classic verification usually focuses on qualitative properties, other applications focus on quantitative properties and models, including probabilistic behaviour and real-time aspects [37]. These applications are usually the results of the evolution of dependability aspects, such as reliability, availability and performance.

Quantitative verification in general includes two main approaches, namely, probabilistic model checking and statistical model checking [38]. Quantitative verification is studied in detail in [37], and this study includes different formalisms, modelling languages, properties and verification approaches. Additionally, different surveys in scientific literature [39,40,41] cover in detail probabilistic model checking, including the main probabilistic models, algorithms and abstraction techniques that describe basic principles and applications.

Some of the available probabilistic modelling tools include: FACT (https://www-users.cs.york.ac.uk/~cap/FACT/ (accessed on 15 October 2022)), MODEST (http://www.modestchecker.net/ (accessed on 15 October 2022)), MRMC (http://mrmc-tool.org/ (accessed on 15 October 2022)), PASS (https://depend.cs.uni-saarland.de/tools/pass/ (accessed on 15 October 2022)), PARAM (https://depend.cs.uni-saarland.de/tools/param/ (accessed on 15 October 2022)), PRISM (http://www.prismmodelchecker.org/ (accessed on 15 October 2022)), UPPAAL (http://www.uppaal.org/ (accessed on 15 October 2022)) and STORM (https://www.stormchecker.org/ (accessed on 15 October 2022)).

The most commonly used tools in the literature are UPPAAL [42,43] and PRISM [44,45], and the newest one is the tool STORM [46,47]. Different probabilistic model-checking tools are analysed and compared in several studies, and a comparison of several tools, including PRISM and STORM, is given in [37], and an extensive comparison between the UPPAAL and PRISM tools is given in [48]. Their authors state that PRISM and STORM tools support the widest range of properties and implemented algorithms, in comparison to other available tools, and that STORM is the most versatile tool. On the other hand, the authors state that due to its extensive online documentation, the graphical user interface and the independence of the platform used, PRISM still represents the standard in research. Since it is also the most commonly used tool, PRISM was selected for the experimental work in this paper.

## 5. Risk and Resilience Assessment for Fraud Alerting Systems

In this section, two system architectures are defined with possible entry points for cyberattacks in the context of an online cloud system. Both proposed fraud alerting systems comprise the same components with the exception of the scoring system, which is present just in one case. For both cases, a risk assessment was conducted in order to identify realistic risks to the system, its resources and data [6]. This work followed the guidelines for conducting risk assessments in the NIST 800-30 standard [49]. According to the risk assessment methodology, first, the threats are identified for the target system. Then, several potential injection points are defined that can be targeted by cyberattacks. Subsequently, several attack scenarios were described for both systems, which depict paths that eventually lead to vulnerability exploitation. For every scenario, exploitation probabilities were calculated from several metrics. In fact, this probability defines the likelihood of a successful exploitation for the given threat. Finally, the outcomes of the modelled attacks are explained, which were generated by one of the modules.

### 5.1. Basic and Extended Fraud Alerting Systems

As already mentioned, this work proposes two conceptual systems for fraud alerting. The basic system, as depicted in Table 3, does not contain a user scoring module, whereas the extended one does. All modules are deployed on separate virtual machines (VM). However, the main module in both systems, namely, the anomaly detection and alerting module, serves as the external entry point. In the context of fintech, this is accessed exclusively by the proper financial institution. Both systems also do data analytics and have a dashboard, which displays the outputs in a human-readable format. Additionally, some modules encompass buffers at their entry points, which represent temporary memories that capture all incoming data. In this context, their main purpose is to receive updates on financial transactions. However, the storage capacity of buffers is not limitless. On the other hand, they are crucial to ensure the functionality of the respective module. In the anomaly detection and alerting module, there also exists a ML component that contains a feature extraction (FE) function. That component processes data from incoming financial transactions in the search for suspicious activity.

The module connects to a database which stores historical data on transactions and attack outcomes from the prior ML algorithm. Needless to say, in order to ensure information security, the database must remain resilient when confronted with cyberattacks. As already noted, this information provides indications in cases of detected issues in transactions.

The extended fraud alerting system contains the scoring system in addition the above mentioned modules, as shown in Figure 2. This means that it also deploys several VMs and a database for alerts. The scoring system encompasses a scoring functionality, which adds another functionality to the fraud alerting system. In cases of correct functionality, the scoring system sends data to an external database, which also keeps track of updates from the anomaly detection and alerting module.

In general, all mentioned components represent possible entry points for cyberattacks in their respective contexts.

### 5.2. Attack Scenarios in Fraud Alerting Systems

In a cloud environment, systems consist of heterogeneous components that interact in a dynamic manner. As such, a deployed system comprises at least one entry point to the external world and its legitimate users. Additionally, the internal components exchange information over predefined communication channels. In such context, cyberattacks can be carried out at multiple injection points separately. However, due to the diversity of such systems, different security vulnerabilities can occur with regard to the target components. Thus, the cloud infrastructure determines the cyberattack surface of the deployed system. For example, databases on the cloud platform are subject to attacks such as SQL Injections (SQLI) [50,51]. Additionally, denial of service (DoS) attacks can be executed in order to disrupt the functionality of an online system [52,53]. Other cyberattacks include man-in-the-middle attacks [54]—eavesdropping especially [55]. However, AI is prone to malicious activity as well. So-called adversarial or backdoor attacks target ML algorithms and models by affecting their processing results [28,56,57].

Due to the distinctive nature of the components, an attacker can carry out different types of attacks. In the proposed fraud alerting systems, the following threats are considered:

- SQL injection [58]: This attack targets the database behind an application and comes in the form of a SQL query. The aim of the attack is to gain access to a database and conduct unauthorized operations on its data entries. In the context of a fraud alerting system, this means that client data can be compromised or modified by an attacker. As a consequence, a fraudulent user or activity may be covered up, or a benign user may become a suspect erroneously.
- Denial of service (DoS) attack [59]: The goal of this type of attack is to interrupt the functionality of a system or to cause access-control restrictions. In this way, the service becomes unavailable for legitimate users or some system component. Typically, successful attacks cause buffer overflows by overflowing the temporary storage with large amounts of data [60]. In addition, this type of attack can come in the form of flooding the target system with network traffic until disabling it altogether [61].
- Adversarial attacks on AI [62]: In the domain of AI, adversarial AI addresses vulnerabilities that can be exploited by ML algorithms. In fact, cyberattacks against ML can disrupt statistical classifiers by injecting malicious data to this algorithm. In such way, malicious data are classified as legitimate during the training phase, whereas legitimate training data are rejected. Thus, typical adversarial attacks include data poisoning, which degrades the performance of the target ML model [28]. In the context of a fraud alerting system, such attacks cause a misclassification of clients with regard to their fraud levels.

**Figure 4:**
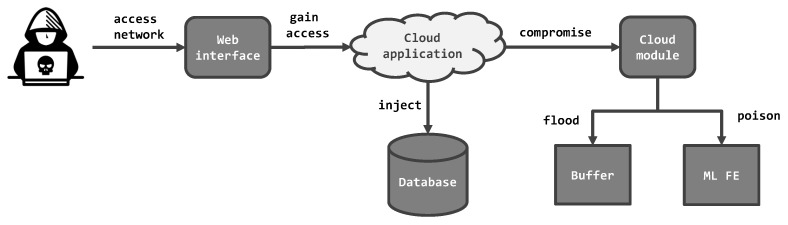
Overview of possible attack scenarios against a cloud-based system.

The threats are assigned to critical assets in the proposed cloud systems, which mark the individual entry points for cyberattacks. Figure 4 depicts possible attack paths of an attacker against the systems in Figure 1 and Figure 2. In this context, several steps can be executed that result in a cyberattack against some component. Basically, a sequence of such steps is considered an attack scenario. Figure 4 covers all possible attack scenarios that can occur in both systems. In each case, the attacker has to gain access to the cloud system over an online network. Afterwards, they aim for a target component, which can be either its internal memory, the ML module or a database. This implies, however, that attacks do not occur sequentially or condition each other. Depending on their choice, the attacker proceeds with one of the above-mentioned attacks. Eventually, the attack culminates in an exploitation and triggers other events, as analysed in Section 4.

The following attack scenarios are defined in the context of the proposed cloud systems. The scenarios correspond to the entry points of their respective systems.

Attack scenario 1

In this scenario, the objective of a cyberattack is to cause an overflow in the buffer of the anomaly detection and alerting module. As a consequence, the posterior ML component will receive corrupted or no information. Theoretically, malicious code can be injected into the buffer, thereby causing damage to the system when executed.

Attack scenario 2

This attack scenario concerns the input module and targets its feature extraction functionality. As a result, incorrect features are derived from adversarial input data, which affects the further fraud alerting process. This happens by increasing significantly the number of false alerts, and potentially masking real frauds by doing so. While adversarial manipulation does not affect the functionality of ML, it does influence the outcomes of its classification process.

Attack scenario 3

In this scenario, an attacker can either insert, update or delete database entries. In the context of the proposed alerting system, the ML-generated message outcomes are stored in a database. In such way, the database becomes subject to this type of attack. In general, a SQLI directly affects the processing outcomes at their back-end.

Attack scenario 4

This attack scenario is the same as Attack Scenario 1 from a technical aspect but differs in its consequences. In this case, a DoS attack is executed against the buffer in the scoring system module. Similarly to the prior example, such attacks can disable further processing in this module. In cases of success, the module can be disabled completely, thereby blocking the user’s scoring functionality. Thus, a staged buffer overflow has an influence on the reputation of a client. This means that decisions on the fraudulent client level can be manipulated, which nullifies the outcome of the previous anomaly detection.

Attack scenario 5

Similarly to the third case, in this scenario, an attacker executes a SQLI against a database. Despite its similarity, this attack follows a different objective. This cyberattack is executed in the context of the extended system against its user scores database. This means that a successful SQLI leads to hacking of client information, which affects users’ reputations from the scoring system. This, in turn, helps an attacker to cover committed frauds and leads the fraud alerting system to provide incorrect information to financial institutions.

### 5.3. Exploitation Probability Assessment

In the next step, the exploitation probabilities for each cyberattack were calculated according to several score metrics. To this end, the already mentioned CVSS was used, which covers multiple characteristics of cyber vulnerability. This framework estimates the severity of a cyberattack and produces a numerical score in form of a probabilistic value. As already mentioned, the score for every cyberattack was determined according to basic vulnerability metrics. These include the proximity of an attacker to their target, the system’s complexity and attack complexity, required user privilege, scope and the implications for information security [63]. Basically, a successful cyberattack at a certain location directly affects the activity and outputs of its posterior component. The National Vulnerability Database (NVD) [64] provides the scores for the CVSS v3.1 calculator, which was used in this work. The base score covers metrics for the attack vector itself, the environment of the target system and the attacker and the impact on information security. Eventually, the probability scores can be consulted to prioritise remediation activities for a cyber vulnerability. It should be noted that the base scores for systems differ only in the number of attacks. That is, the extended fraud alerting system encompasses two additional attacks, whereas the base scores of common ones remain the same.

However, it should be noted that some assumptions are valid for the risk assessment of both systems. First, it was assumed that an attacker had gained access to a network that was connected to the cloud system. Additionally, all target components were considered to be vulnerable, which means that they could be exploited. The assumption was that the attacker possessed sufficient domain knowledge and hacking skills in order to execute the mentioned attacks. It is important to note that the goal was not to compromise the system but to analyse the risks of particular attacks and their impact.

The context of both DoS attacks, the attacker gains access to the target network and causes flooding to disable the target buffer. Eventually, no special user privilege and no additional user interaction is required for this attack. The exploited vulnerability’s scope remains unchanged with some loss of confidentiality and integrity. In this attack, the attacker is fully able to violate availability of the target service; thus, the base score equals 8.6. The target component for an adversarial attack is bound to the network stack and requires user privileges. In cases of success, this attack violates information confidentiality and integrity but does not affect its availability. The score for the adversarial attack equals 6.1. An SQLI in the given contexts does not require special physical proximity nor advanced attacker skills. However, in case of success, it does affect information security to a certain degree and results in a base score of 6.5.

The base scores for all mentioned side-channel and adversarial attacks against the basic system are given in Table 3. As can be seen, the scores for DoS, poisoning and SQLI retain the same values due to the fact that each attack scenario is considered separately. For this reason, all common attacks have the same traits in the metric system. Due to the relative low complexity of these attacks, DoS has a higher base score, whereas the more complex attacks encompass lower values.

In the extended fraud alerting system, additional cyberattacks add two more values to the total base score, as presented in Table 4. Additionally, attacks of the same type have equal values in their given context. As was the case with its basic counterpart, the extended system contains one attack each for DoS, adversarial manipulation and SQLI. Additionally, the additional SQLI and DoS attacks share the same scores as in the basic system. However, despite these facts, the two additional attacks increase the total score value.

### 5.4. Modelled Attack Outcomes

The outcomes of the previously selected cyberattacks were identified and modelled for the proposed systems in the following manner:

- Missed alert—transaction was fraudulent but the system did not detect it,
- False alert—transaction was regular, but system created an alert.

Both outcomes were formally checked in two cases. In the first, no active attacks were present in the system. In the second, an attacker actively approached the system and possessed the means and knowledge to perform a successful attack. The previously described exploitation probabilities reflect the likelihood that an attack against a certain component will be successful. However, the attacker’s capability here was limited to the maximum number of two attacks against different components during the system’s run.

The modelled attack outcomes are described in detail in Table 5, including the initial and final states for both systems. It should be noted that state Fraud indicates whether the transaction is fraudulent (Fraud=1) or not (Fraud=0). Alert states indicate if fraud is suspected ($Alert_{anomaly}$ / $Alert_{score}$ = 1) by the system or not, at a given moment, for a given transaction. It should also be noted that both missed and false alerts in the extended system are defined through both indicators having the same value (e.g., $Alert_{anomaly}=0$ and $Alert_{score}=0$). On the other hand, the cases where indicators show different results should receive special treatment, depending on the exact application requirements, and could be seen as a form of the “light” alert.

## 6. Results and Discussion

The selected model checker, PRISM, was applied on two proposed fraud detection system architectures—basic and extended, in order to perform a formal risk and resilience analysis and to obtain an indication on how safety and security requirements are fulfilled within a given environment. Subsequently, the modelled attack scenarios were selected from the scenarios outlined in Section 5. This section describes the results of formal modelling and the resilience assessment analysis in form of risk-exposure scores, and discusses their implications.

### 6.1. Parallel Detectors’ Influence on the Detection Performance

The first round of tests show the anomaly detection and alerting module’s behaviour in the case when no active cyberattacks are present. Nevertheless, potential financial frauds are considered in form of a fraud probability, which reflects the expected user behaviour.

The anomaly detection and alerting module is modelled in the form of a non-deterministic system model, namely, a Markov decision process (MDP). The system’s architecture, attacker’s behaviour and existing vulnerabilities in the system, including their identified exploitation probabilities, are incorporated into the Markov model. Formal modelling and checking of the system is performed by using the PRISM model checker. This results in the system model, the identified attack properties and risk-exposure scores as quantifiable risk and resilience indicators.

The conducted tests indicate that the use of three parallel anomaly detectors, with the logic described in Section 3, results in a positive impact on the detection performance. This is measured in true positive rate and true negative rate. Table 6 quantifies the performance and compares it to the given ML detectors [33] used separately (as the only anomaly-detection logic in the system). According to the results, it is obvious that parallel detectors significantly outperform separate methods under given assumptions, measured through both TPR and TNR.

### 6.2. Cyber-Attacks’ Influence on the System’s Performance

As previously stated, it was assumed during the modelling process that an attacker actively tries to exploit the system and possesses the necessary skills and knowledge. In this case, the maximum number of vulnerabilities that can be exploited in one attack scenario—the cost value, measures an attacker’s skills. In this experiment, the cost value is set to two, which indicates a fairly skilled attacker. In addition, it is assumed that the attacker is aware of the availabilities from Section 5. For this sake, at least one exploitation is necessary for a successful attack attempt. The model also implies that during one iteration the choice of a target vulnerability is random. Additionally, corresponding exploitation probabilities are defined for all listed vulnerabilities. These values indicate the likelihood that a particular vulnerability will be successfully exploited during an attempt, under previously given assumptions.

During the next step, attack properties are defined and added to the completed system model. In general, such properties define formal definitions and preconditions for a successful attack attempt within the modelled system. The attack properties are defined by using the probabilistic computation tree logic (PCTL), which is embedded in the PRISM model checker. The subsequent formal check results in the maximum likelihood of a successful attack attempt—the risk-exposure scores. All modelled attack properties are directly based on the given attack outcomes, as described in Table 5. In addition, Table 7 shows the obtained values for risk-exposure scores, under the previously described assumptions.

The obtained results show the maximum likelihood of a successful attack attempt, by considering different system architectures. Assuming that the goal of an attacker is to carry out an attack by exploiting the smallest amount of vulnerabilities, the presented analysis considers cases where the maximum number of exploited vulnerabilities is set to two.

The results also show that an attack against the basic system architecture has a high likelihood to cause either missed or false alerts, where the system is more susceptible to missed alerts. Adding additional decision logic in form of the proposed scoring system has a positive impact on the system’s resilience. This is the case both when the system is under attack, and in a case of uninterrupted system operation. This effect is especially important when it comes to false alerts boosted by active cyberattacks (reduced from 0.8612 to 0.5669/0.6315, depending on the threshold selection), but is also indicted through decrease in missed alert likelihood under the same conditions (reduced from 0.9524 to 0.8236/0.7519). Nevertheless, the positive effect is also present in the case of regular system operation, without cyberattacks, indicated through a decrease in false alerts (0.0288 to 0.0007/0.0143) and a slight decrease in missed alerts (0.0291 to 0.0291/0.0281).

The scoring system threshold has also a certain impact on the results—setting the threshold value to 1⁄2 causes a greater false-alert likelihood decrease; setting it to 2⁄3 causes a greater missed-alerts likelihood. The final choice should depend on the exact application requirements.

Finally, as previously noted, both missed and false alerts in the extended system are defined as "strong" alerts (e.g., $Alert_{anomaly}\;\&\;Alert_{score}=0/1$). The situations where indicators show different results could be regarded as a form of "light" alert, and should be specially defined and treated depending on the exact application requirements and environment.

## 7. Conclusions and Future Work

In this paper, an important security issue in modern fintech applications is addressed. Emerging technological advances in ubiquitous and cloud computing offer unforeseen functionality and do not come without drawbacks. For example, fighting financial and cyber crime remains an important challenge for cybersecurity in this environment. For this sake, a conceptual architecture is introduced that describes an alerting system for the early detection of a common type of fraud, namely, credit card theft. In fact, the proposed system represents a fintech cloud service, which is consulted by a financial institution to identify malicious users in credit card transactions. After defining the structure of the fraud alerting system, a risk assessment is conducted to address potential security issues. Therefore, cyber threats are identified that endanger the critical components in the system. Potential vulnerabilities are identified, whose exploitation probability is subsequently determined according to several base metrics. In addition to the design of its architecture, a formal model of the system was made in PRISM, a state-of-the-art modelling language. The resulting formal model includes cyber threats and exploitation likelihoods and defines additional security properties that might affect the cloud system. Subsequently, the model checker is used to derive risk exposure rates for the corresponding attacks.

The goal of the system is to provide an efficient and automated way to detect frauds in financial transaction data records. This process is further augmented by applying different ML techniques for anomaly detection. Subsequently, the efficiency of the system was addressed in terms of parallel processing, behavioural analysis and its robustness. As a result, the influences of individual cyberattacks and parallel anomaly detectors on the performance was analysed in particular. The initial results imply that parallel processing brings significant benefits to the overall detection rates and increases the resilience of the system as a whole. Additionally, introducing the additional alerting—i.e., scoring—module, contributes to the resilience of the alerting systems. In fact, it increases the security level of the considered cloud service, both in cases of regular system operation (not interrupted by cyberattacks) and in cases when the system is subject to active cyberattacks.

The promise of the presented concept is its applicability to real-world environments. Additionally, it provides an overview of potential cybersecurity issues that must be considered when implementing a fraud alerting system. The proposed analysis considered realistic challenges to ensure correct and cyber secure functionality before its implementation. To our knowledge, the proposed solution represents one of the earliest contributions to the mentioned challenges. In this regard, the provided formal model constitutes an early specification for this type of system. As such, the proposed solution further contributes to security-by-design of anomaly detection systems in the fintech domain. In the future, the proposed architecture will be further extended by broadening the risk assessment in terms of cyber threats and vulnerabilities. Additional security properties that mirror realistic threats will be considered for formal specification. Finally, the produced knowledge will be useful for other disciplines, especially for the domain of anomaly detection and security testing.

## Figures and Tables

**Table 2:** Performance of the selected outlier detection methods [33].

	Outlier Detection Method	TPR	TNR
ML1	Local Outlier Factor	0.8824	0.8960
ML2	Isolation Forest	0.9265	0.8992
ML3	Elliptic Envelope	0.8824	0.9003

**Table 3:** Exploitation probability scores for the basic system.

#	Type of Attack	Affected Component (Domain)	Base Score
1	Denial-of-Service	Buffer (Anomaly detection and alerting module)	8.6 (High)
2	Adversarial attack	Feature Extraction module	6.1 (Medium)
	(poisoning)	(Anomaly detection and alerting module)	
3	SQL injection	Alerts database	6.5 (Medium)

**Table 4:** Exploitation probability scores for the extended system.

#	Type of Attack	Affected Component (Domain)	Base Score
1	Denial-of-Service	Buffer (Anomaly detection and alerting module)	8.6 (High)
2	Adversarial attack	Feature Extraction module	6.1 (Medium)
	(poisoning)	(Anomaly detection and alerting module)	
3	Denial-of-Service	Buffer (Blacklisting module)	8.6 (High)
4	SQL injection	Alerts database	6.5 (Medium)
5	SQL injection	Scores database	6.5 (Medium)

**Table 5:** Possible outcomes of the modelled attacks.

			Final State	
Outcome	Description	Initial State	Basic System	Extended System
Missed alert (no attacks)	Transaction was fraudulent but the system did not detect it during uninterrupted system operation	Fraud=1	$Alert_{anomaly}=0$	$Alert_{anomaly}=0$ & $Alert_{score}=0$
Missed alert (active attacks)	Transaction was fraudulent but the system did not detect it during system operation potentially interrupted by cyber-attacks	Fraud=1	$Alert_{anomaly}=0$	$Alert_{anomaly}=0$ & $Alert_{score}=0$
False alert (no attacks)	Transaction was regular, but system created an alert during uninterrupted system operation	Fraud=0	$Alert_{anomaly}=1$	$Alert_{anomaly}=1$ & $Alert_{score}=1$
False alert (active attacks)	Transaction was regular, but system created an alert during system operation potentially interrupted by cyber-attacks	Fraud=0	$Alert_{anomaly}=1$	$Alert_{anomaly}=1$ & $Alert_{score}=1$

**Table 6:** Resulting performance of the parallel anomaly detection process.

	Outlier Detection Method	TPR	TNR
** ML1∥ML2∥ML3 **	Proposed parallel processing	0.9709	0.9712
ML1	Local Outlier Factor	0.8824	0.8960
ML2	Isolation Forest	0.9265	0.8992
ML3	Elliptic Envelope	0.8824	0.9003

**Table 7:** Resulting risk-exposure scores for the system under normal operation and under active cyberattacks.

		Extended System	
Outcome	Basic System	Threshold=1⁄2	Threshold=2⁄3
Missed alert (no attacks)	0.0291	0.0291	0.0281
Missed alert (active attacks)	0.9524	0.8236	0.7519
False alert (no attacks)	0.0288	0.0007	0.0143
False alert (active attacks)	0.8612	0.5669	0.6315

## Data Availability

Not applicable.

## References

[1] Bettinger A. (1972). FINTECH: A Series of 40 Time Shared Models Used at Manufacturers Hanover Trust Company. Interfacec.

[2] Thakor A.V. (2020). Fintech and banking: What do we know?. J. Financ. Intermediation.

[3] Lynn T., Mooney J.G., Rosati P., Cummins M. Disrupting finance: FinTech and strategy in the 21st century. Proceedings of the International Conference on Artificial Intelligence and Computer Vision (AICV2020), Advances in Intelligent Systems and Computing.

[4] Vivek D., Rakesh S., Walimbe R.S., Mohanty A. (2020). The Role of CLOUD in FinTech and RegTech. Ann. Dunarea Jos Univ. Galati-Fascicle Econ. Appl. Inform..

[5] Microsoft Azure: Cloud Computing Services. https://azure.microsoft.com.

[6] Kott A., Linkov I. (2019). Cyber Resilience of Systems and Networks.

[7] Dal Pozzolo A., Boracchi G., Caelen O., Alippi C., Bontempi G. (2017). Credit card fraud detection: A realistic modeling and a novel learning strategy. IEEE Trans. Neural Netw. Learn. Syst..

[8] Kaur G., Habibi Lashkari Z., Habibi Lashkari A. (2021). Cybersecurity Threats in FinTech. Underst. Cybersecur. Manag. Fintech. Future Bus. Financ..

[9] Martins N., Magalhães Cruz J., Cruz T., Abreu P.H. (2020). Adversarial Machine Learning Applied to Intrusion and Malware Scenarios: A Systematic Review. IEEE Access.

[10] Imerman M., Patel R., Kim Y.D. (2022). Cloud finance: A review and synthesis of cloud computing and cloud security in financial services. J. Financ. Transform. Capco Inst..

[11] Kettani H., Cannistra R.M. On Cyber Threats to Smart Digital Environments. Proceedings of the 2nd International Conference on Smart Digital Environment (ICSDE’18).

[12] Tsaregorodtsev A.V., Kravets O.J., Choporov O.N., Zelenina A.N. (2018). Information Security Risk Estimation for Cloud Infrastructure. Int. J. Inf. Technol. Secur..

[13] Common Vulnerability Scoring System SIG. https://www.first.org/cvss.

[14] Sun X., Liu P., Singhal A. (2018). Toward Cyberresiliency in the Context of Cloud Computing. IEEE Secur. Priv..

[15] Furfaro A., Piccolo A., Parise A., Argento L., Saccà D. (2018). A Cloud-based platform for the emulation of complex cybersecurity scenarios. Future Gener. Comput. Syst..

[16] Singh Sohal A., Sandhu R., Sood S.K., Chang V. (2018). A cybersecurity framework to identify malicious edge device in fog computing and cloud-of-things environments. Comput. Secur..

[17] Hawasli A. (2018). AzureLang: A Probabilistic Modeling and Simulation Language for Cyber Attacks in Microsoft Azure Cloud Infrastructure. Master’s Thesis.

[18] Sontowski S., Gupta M., Chukkapalli S.S.L., Abdelsalam M., Mittal S., Joshi A., Sandhu R. Cyber Attacks on Smart Farming Infrastructure. Proceedings of the International Conference on Collaborative Computing: Networking, Applications and Worksharing (CollaborateCom).

[19] Jauhiainen H. (2018). Designing End User Area Cybersecurity for Cloud-Based Organization. Master’s Thesis.

[20] MITRE ATT&CK®. https://attack.mitre.org.

[21] Sabur A., Chowdhary A., Huang D., Alshamrani A. (2022). Toward scalable graph-based security analysis for cloud networks. Comput. Netw..

[22] George G., Thampi S.M. (2019). Vulnerability-based risk assessment and mitigation strategies for edge devices in the Internet of Things. Pervasive Mob. Comput..

[23] Souaf S., Berthomó P., Loulergue F. A Cloud Brokerage Solution: Formal Methods Meet Security in Cloud Federations. Proceedings of the 2018 International Conference on High Performance Computing & Simulation (HPCS).

[24] Gomes Valadares D., Alvares de Carvalho César Sobrinho A., Perkusich A., Costa Gorgonio K. (2021). Formal Verification of a Trusted Execution Environment-Based Architecture for IoT Applications. IEEE Internet Things J..

[25] Waqas M., Tu S., Halim Z., Ur Rehman S., Abbas G., Haq Abbas Z. (2022). The role of artificial intelligence and machine learning in wireless networks security: Principle, practice and challenges. Artificial Intelligence Review.

[26] Al Nafea R., Almaiah M.A. Cyber Security Threats in Cloud: Literature Review. Proceedings of the International Conference on Information Technology (ICIT).

[27] Ahmad W., Rasool A., Javed A.R., Baker T., Jalil Z. (2022). Cyber Security in IoT-Based Cloud Computing: A Comprehensive Survey. Electronics.

[28] Duddu V. (2018). A Survey of Adversarial Machine Learning in Cyber Warfare. Def. Sci. J..

[29] Alt F. (2021). Pervasive Security and Privacy—A Brief Reflection on Challenges and Opportunities. IEEE Pervasive Comput..

[30] Kulik T., Dongol B., Larsen P.G., Macedo H.D., Schneider S., Tran-Jorgensen P.W.V., Woodcock J. (2022). A Survey of Practical Formal Methods for Security. Form. Asp. Comput..

[31] Tissir N., El Kafhali S., Aboutabit N. (2021). Cybersecurity management in cloud computing: Semantic literature review and conceptual framework proposal. J. Reliab. Intell. Environ..

[32] Vallant H., Stojanović B., Božić J., Hofer-Schmitz K. (2021). Threat Modelling and Beyond-Novel Approaches to Cyber Secure the Smart Energy System. Appl. Sci..

[33] Stojanović B., Božić J., Hofer-Schmitz K., Nahrgang K., Weber A., Badii A., Sundaram M., Jordan E., Runevic J. (2021). Follow the trail: Machine learning for fraud detection in Fintech applications. Sensors.

[34] PRISM—Probabilistic Symbolic Model Checker. https://www.prismmodelchecker.org.

[35] Keerthi K., Roy I., Hazra A., Rebeiro C. (2019). Formal verification for security in IoT devices. Secur. Fault Toler. Internet Things.

[36] Basin D., Cremers C., Meadows C. (2018). Model checking security protocols. Handbook of Model Checking.

[37] Hahn E.M., Hartmanns A., Hensel C., Klauck M., Klein J., Křetínskỳ J., Parker D., Quatmann T., Ruijters E., Steinmetz M. (2019). The 2019 comparison of tools for the analysis of quantitative formal models. International Conference on Tools and Algorithms for the Construction and Analysis of Systems.

[38] Hofer-Schmitz K., Stojanović B. (2020). Towards formal verification of IoT protocols: A Review. Comput. Netw..

[39] Katoen J.P. The probabilistic model checking landscape. Proceedings of the 31st Annual ACM/IEEE Symposium on Logic in Computer Science.

[40] Bartels F., Sokolova A., de Vink E. (2004). A hierarchy of probabilistic system types. Theor. Comput. Sci..

[41] Hartmanns A., Hermanns H. (2015). In the quantitative automata zoo. Sci. Comput. Program..

[42] Bengtsson J., Larsen K., Larsson F., Pettersson P., Yi W. (1995). UPPAAL—A tool suite for automatic verification of real-time systems. International Hybrid Systems Workshop.

[43] Behrmann G., David A., Larsen K.G. (2006). A Tutorial on Uppaal 4.0..

[44] Hinton A., Kwiatkowska M., Norman G., Parker D. (2006). PRISM: A tool for automatic verification of probabilistic systems. Proceedings of the International Conference on Tools and Algorithms for the Construction and Analysis of Systems.

[45] Kwiatkowska M., Norman G., Parker D. (2011). PRISM 4.0: Verification of probabilistic real-time systems. Proceedings of the International Conference on Computer Aided Verification.

[46] Dehnert C., Junges S., Katoen J.P., Volk M. (2017). A storm is coming: A modern probabilistic model checker. Proceedings of the International Conference on Computer Aided Verification.

[47] Hensel C., Junges S., Katoen J.P., Quatmann T., Volk M. (2022). The probabilistic model checker Storm. Int. J. Softw. Tools Technol. Transf..

[48] Naeem A., Azam F., Amjad A., Anwar M.W. (2018). Comparison of model checking tools using timed automata-PRISM and UPPAAL. Proceedings of the 2018 IEEE International Conference on Computer and Communication Engineering Technology (CCET).

[49] (2012). Guide for Conducting Risk Assessments. https://www.proquest.com/openview/18c4c4b072ef4af28d2bf91db8e278b8/1?pq-origsite=gscholar&cbl=41798.

[50] Tripathy D., Gohil R., Halabi T. Detecting SQL Injection Attacks in Cloud SaaS using Machine Learning. Proceedings of the International Conference on Big Data Security on Cloud (BigDataSecurity), High Performance and Smart Computing (HPSC) and Intelligent Data and Security (IDS).

[51] Xiao F., Zhijian W., Meiling W., Ning C., Yue Z., Lei Z., Pei W., Xiaoning C. (2021). An old risk in the new era: SQL injection in cloud environment. Int. J. Grid Util. Comput..

[52] Gupta B.B., Badve O.P. (2017). Taxonomy of DoS and DDoS attacks and desirable defense mechanism in a Cloud computing environment. Neural Comput. Appl..

[53] Somani G., Singh Gaur M., Sanghi D., Conti M., Buyya R. (2017). DDoS attacks in cloud computing: Issues, taxonomy, and future directions. Comput. Commun..

[54] Logesswari S., Jayanthi S., KalaiSelvi D., Muthusundari S., Aswin V. (2020). A study on cloud computing challenges and its mitigations. Mater. Today Proc..

[55] Santoso L.W. Cloud Technology: Opportunities for Cybercriminals and Security Challenges. Proceedings of the Twelfth International Conference on Ubi-Media Computing (Ubi-Media).

[56] Chen Y., Gong X., Wang Q., Di X., Huang H. (2020). Backdoor Attacks and Defenses for Deep Neural Networks in Outsourced Cloud Environments. IEEE Netw..

[57] Ma Z., Ma J., Miao Y., Liu X., Choo K.K.R., Deng R.H. (2021). Pocket Diagnosis: Secure Federated Learning against Poisoning Attack in the Cloud. IEEE Trans. Serv. Comput..

[58] SQL Injection. https://owasp.org/www-community/attacks/SQL_Injection.

[59] Denial of Service. https://owasp.org/www-community/attacks/Denial_of_Service.

[60] Buffer Overflow Attack. https://owasp.org/www-community/attacks/Buffer_overflow_attack.

[61] Understanding Denial-of-Service Attacks. https://www.cisa.gov/uscert/ncas/tips/ST04-015.

[62] Vorobeychik Y., Kantarcioglu M. (2018). Adversarial Machine Learning.

[63] Common Vulnerability Scoring System Version 3.1 Calculator. https://www.first.org/cvss/calculator/3.1.

[64] National Vulnerability Database. https://nvd.nist.gov.

